# Pathological insights from amyotrophic lateral sclerosis animal models: comparisons, limitations, and challenges

**DOI:** 10.1186/s40035-023-00377-7

**Published:** 2023-09-20

**Authors:** Longhong Zhu, Shihua Li, Xiao-Jiang Li, Peng Yin

**Affiliations:** Guangdong Key Laboratory of Non-Human Primate Research, Key Laboratory of CNS Regeneration (Ministry of Education), GHM Institute of CNS Regeneration, Jinan University, Guangzhou, 510632 China

**Keywords:** Amyotrophic lateral sclerosis, Animal model, Rodent, Non-human primate

## Abstract

In order to dissect amyotrophic lateral sclerosis (ALS), a multigenic, multifactorial, and progressive neurodegenerative disease with heterogeneous clinical presentations, researchers have generated numerous animal models to mimic the genetic defects. Concurrent and comparative analysis of these various models allows identification of the causes and mechanisms of ALS in order to finally obtain effective therapeutics. However, most genetically modified rodent models lack overt pathological features, imposing challenges and limitations in utilizing them to rigorously test the potential mechanisms. Recent studies using large animals, including pigs and non-human primates, have uncovered important events that resemble neurodegeneration in patients’ brains but could not be produced in small animals. Here we describe common features as well as discrepancies among these models, highlighting new insights from these models. Furthermore, we will discuss how to make rodent models more capable of recapitulating important pathological features based on the important pathogenic insights from large animal models.

## Introduction

Amyotrophic lateral sclerosis (ALS), also known as Lou Gehrig’s disease, is a classical neurodegenerative disease characterized by progressive degeneration of both upper and lower motor neurons in the brain and spinal cord [1]. ALS is divided into sporadic (sALS) and familial (fALS) forms based on heredity, with approximately 5%–10% of cases being fALS [2]. The onset of ALS usually occurs at late middle ages, and the clinical manifestations are progressive muscle atrophy and weakness. Most patients will die of respiratory failure within 2–5 years [3]. Whole-exome and whole-genome sequencing has identified fALS-associated mutations in approximately 50 genes, and more than 30 are considered causative genes [4]. The most commonly mutated genes are superoxide dismutase-1 (*SOD1*) with a mutation frequency of 25% in fALS cases (the wild-type [WT] or misfolded SOD1 has also been implicated in a significant fraction of sALS cases) [5], chromosome 9 open reading frame 72 (*C9ORF72*) with a mutation frequency of 39% in fALS cases and 7% in sALS cases [6–8], fused in sarcoma (*FUS*) with a mutation frequency of 2.8% in fALS cases and 0.3% in sALS cases [9–11], and TAR DNA-binding protein (*TARDBP*) with a mutation frequency of 4.2% in fALS cases and 0.8% in sALS cases [11, 12]. Most of the current ALS models are based on these four genes, including both vertebrate and invertebrate models such as yeast, elegans, fruit flies, zebrafish, mice, rats, dogs, pigs, and more recently, non-human primates. These models have different characteristics and are complementary in the dissection of pathological mechanisms underlying motor neuron degeneration and ALS progression. However, most transgenic small animal models lack significant neurodegenerative features compared to large animal models, presenting challenges and limitations in their use [13]. In this review, the four genes currently most associated with ALS prevalence are discussed, with a focus on evidence derived from ALS patients. Then we describe common features as well as discrepancies among these models, highlighting new insights and emerging roles of experimental organisms in ALS research.

## ALS genes in ALS patients

### *SOD1*

The *SOD1* gene encodes a ubiquitous Cu/Zn superoxide dismutase that catalyzes the dismutation of superoxide radicals into hydrogen peroxide and dioxygen. Rosen et al. [14] first reported the genetic link between fALS and the *SOD1* gene in 1993. Teepu Siddique et al. [15] subsequently reported a breakthrough finding that dominant, gain-of-function mutations in *SOD1* contribute to the pathogenesis of fALS. In the ensuing two decades, more than 185 disease-associated *SOD1* variants were identified, distributed throughout the gene [16]. Genotype–phenotype correlations are evident in *SOD1*-ALS, with distinct clinical features in patients harboring specific variants [17]. Globally, the most common mutation in the *SOD1* gene is D90A, and carriers of this mutation typically present with a slowly progressive paralysis that begins in the legs and spreads upwards, with atypical features such as bladder dysfunction [18]. Mutations in *SOD1* are found in approximately 20% of fALS patients, but the mechanism by which mutant *SOD1* triggers motor neuron damage remains controversial. Currently, more evidence supports that the mutation-induced SOD1 conformational and functional changes confer toxicity through interactions with many proteins and cause a series of consequences, including excitotoxicity, endoplasmic reticulum stress, oxidative stress through upregulation of reactive oxygen species, mitochondrial dysfunction, and prion-like proliferation [19]. The fact that *SOD1*-knockout mice also develop muscle denervation and mitochondrial oxidative stress over time suggests that chronic loss of SOD1 activity may contribute to disease [20, 21].

### *TARDBP*

TDP-43, encoded by the *TARDBP* gene, is a DNA/RNA-binding protein consisting of 414 amino acids. TDP-43 contains nuclear localization signals and nuclear export signals, allowing it to shuttle between the nucleus and the cytoplasm, but typically it resides in the nucleus to exert important functions such as gene regulation [22]. More than 50 missense mutations in the *TARDBP* gene have been identified in ALS patients, accounting for 1%–2% of the total cases [23]. TDP-43 has been reported to be involved in several RNA processing steps, including pre-mRNA splicing, mRNA transport, regulation of mRNA stability, translation, and regulation of non-coding RNAs [24]. In 2006, TDP-43 was identified as a key component of neuronal and glial cytoplasmic inclusions in patients with ALS and frontotemporal lobar degeneration (FTLD or FTLD-TDP) [25, 26]. These inclusion bodies are mainly aggregates of pathological TDP-43 proteins that are hyperphosphorylated, ubiquitinated, and cleaved at the C-terminus. Pathological TDP-43 protein aggregation is often accompanied by loss of nuclear TDP-43, suggesting a loss of normal function in the nucleus, increased abnormal cytoplasmic function, or both [27]. The accumulation of TDP-43 in ubiquitin-positive cytoplasmic neuronal inclusions in the brain and spinal cord has been recognized as a pathological hallmark of ALS [28].

### *FUS*

In 2009, pathogenic variants in the gene encoding FUS, another TDP-43-like RNA-binding protein, were reported in patients with ALS [9]. FUS is a ubiquitously expressed 526-amino-acid protein, which shares many physiological roles with TDP-43 in various aspects of gene expression and is involved in several RNA processing events, in particular transcription, alternative splicing, and mRNA trafficking. FUS is predominantly localized to the nucleus under normal physiological conditions, but it crosses the nuclear membrane to play a role in nucleoplasmic transport, similar to TDP-43 [29]. More than 50 autosomal-dominant *FUS* variants have been identified in ALS patients [30]. Many of these mutations affect the nuclear localization of FUS, leading to loss of function as a regulator of transcription and RNA maturation, and formation of toxic FUS aggregates in the cytoplasm [31–34]. Therefore, the pathogenic mechanism of FUS is similar as TDP-43, involving combined loss of function and toxic aggregation. In addition, FUS is also involved in DNA repair mechanisms, including strand-break repair, non-homologous end joining, and homologous recombination during DNA binuclear recombination [35].

### *C9ORF72*

The *C9ORF72* gene contains 12 exons and encodes a small protein that plays a key role in the regulation of autophagy. Crystal structures and biochemical analysis of purified recombinant proteins support a role for the C9ORF72 complex as a GTPase-activating protein [36–38]. In 2011, expansion of a hexanucleotide repeat (GGGGCC) in the noncoding region of *C9ORF72* was reported to be the most common genetic cause of ALS in European populations [39]. There are 5–10 copies of the hexanucleotide repeat in the normal *C9ORF72* gene, but the number of repeats may increase to hundreds to thousands in ALS patients. This hexanucleotide repeat expansion is found in approximately 34% of fALS and 5% of sALS cases in European populations but occurs less frequently in Asian populations [11]. The pathogenic role of *C9ORF72* is still controversial, but increasing evidence indicates that the pathogenic mechanism of *C9ORF72*-ALS involves a cascade of reactions, including multiple cellular mechanisms: (1) G4C2 hexanucleotide repeat expansion causes RNA toxicity [40, 41]; (2) aggregation of toxic dipeptide repeat proteins (DPRs) translated from the hexanucleotide repeats through repeat-associated non-ATG translation [42, 43]; and (3) decreased levels of normally functioning C9ORF72 protein, leading to loss-of-function mechanisms [44]. There is no consensus on the extent to which each of these mechanisms contributes to disease progression, but all of them could explain the pathogenic role of the hexanucleotide expansion in *C9ORF72*.

**Fig. 1:**
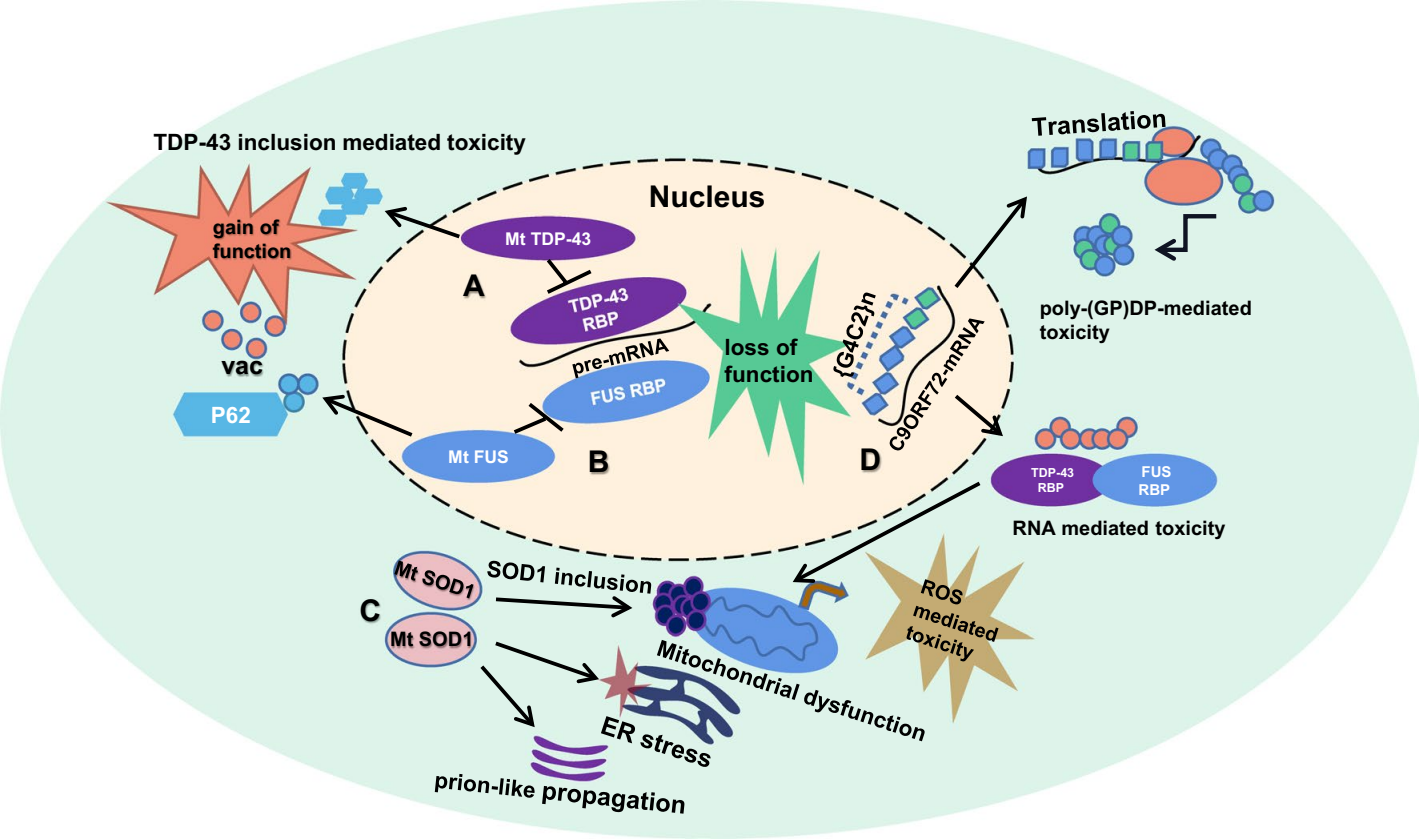
Pathogenesis of *TARDBP*-, *FUS*-, *SOD1*- and *C9OR72*-associated ALS. **a** *TARDBP* mutations act through both loss-of-function and gain-of-function mechanisms. Mutant (Mt) TDP-43 proteins inhibit normal TDP-43 binding to pre-mRNA and generate TDP-43 inclusions in the cytoplasm. **b** Like TDP-43, *FUS* mutations act through both loss-of-function and gain-of-function mechanisms. Mutant FUS proteins cause loss-of-function by inhibiting normal FUS binding to pre-mRNA. **c** *SOD1* mutations act through a gain-of-function mechanism. Mutant SOD1 dimer accumulation in the cytoplasm leads to prune-like toxin production and Golgi apparatus stress, and generation of mitochondrial reactive oxygen species (ROS), leading to mitochondrial destruction. **d** The *C9ORF72* mutation acts through a gain-of-function mechanism. GGGGCC[G4C2] translocates to the cytosol, translates to form aggregates of poly (GP) DPRs, and misfolds to form aggregates of ubiquitinated RNA aggregates associated with TDP-43 or FUS proteins. Both proteins mediate neuronal toxicity. Cytosol vacuolization (vac) is caused by all the above-mentioned mutations

The specific pathogenic mechanism of the four genes are illustrated in Fig. 1. Apart from these genes, other less common genetic mutations have also been reported to be associated with ALS, such as mutations in *OPTN* (Optineurin), *VABP* (VAMP-associated protein B), *UBQLN2* (Ubiquilin-2), *VCP* (Valosin containing protein), *MATR3* (Matrin 3), *TBK1* (TANK-binding kinase-1), *NEK1* (NIMA-related kinase-1), and *C21orf2* [45]. In addition, the interactions between environmental factors and genetic mutations must also be considered.

## Models for ALS

### *Caenorhabditis elegans* and Zebrafish (*Danio rerio*)

The anatomical transparency of *C. elegans* and Zebrafish enables visualization of neurons and monitoring of neuronal activity over time using co-expressed fluorescent proteins. In addition, the well-defined and genetically controllable nervous system of *C. elegans* and Zebrafish is also an advantage for exploring the pathological mechanisms of neurodegenerative diseases and provides a good tool for screening new potential drugs [46–49].

#### *Caenorhabditis elegans* and Zebrafish models of SOD1 mutation

In a *C. elegans* SOD1 model, overexpression of human *SOD1* (G85R) in neurons results in cytoplasmic aggregates, reduced number and diameter of cellular processes, reduced number of mitochondria and vesicles, and motility deficits. *Caenorhabditis elegans* overexpressing human *SOD1* (H46R or H48Q) also exhibits motor deficits, but to a lesser extent than those overexpressing *SOD1* (G85R). Overexpression of human *SOD1* (G93A) in motor neurons leads to age-dependent paralysis due to axonal defects, but no overt neuronal death was observed [50, 51]. Injection of human *SOD1* (G93A, G37R or A4V) mRNA into zebrafish embryos resulted in abnormal axonal branching and shortened axon length, and these phenotypes were aggravated when the models expressed higher levels of mutant *SOD1* [52, 53]. Furthermore, in zebrafish expressing mutant SOD1, the motor impairment, protein misfolding, and ER-Golgi transport dysfunction were rescued by protein disulfide isomerase, suggesting that redox regulation is essential for maintaining cellular homeostasis [54].

#### *Caenorhabditis elegans* and Zebrafish models of FUS mutation

Expression of *FUS* variants in *C. elegans* induces protein aggregation in GABAergic neurons, leading to neurodegeneration, synaptic dysfunction, and paralysis, together with cytoplasmic mislocalization of FUS, whereas expression of WT *FUS* does not cause significant changes [55, 56]. Furthermore, electron microscopy and electrophysiological analysis showed that transgenic expression of mutant *FUS* in *C. elegans* resulted in reduced synaptic transmission from motor neurons to muscles and impaired synaptic vesicle docking at the neuromuscular junction (NMJ) [57]. In zebrafish models, knockdown of endogenous FUS or overexpression of human defective *FUS* alleles leads to defective presynaptic function at the NMJ, producing pathological motor phenotypes that can be rescued by co-expression of WT human FUS [58]. Additional studies indicate that *FUS* mutations in zebrafish also induce protein aggregation, oxidative stress, NMJ damage, and motor dysfunction in motor neurons (MN) and other cells [59–62].

#### *Caenorhabditis elegans* and Zebrafish models of TDP-43 mutation

Ash et al. developed the first TDP-43 overexpression model in *C. elegans* and showed that pan-neuronal expression of human WT TDP-43 in transgenic *C. elegans* resulted in movement disorders, as well as fasciculations of motor neurons [63, 64]. Furthermore, expression of mutant TDP-43 in nematode GABAergic motor neurons induces oxidative stress and aberrant expression of endogenous TDP-1 (an ortholog of TARDBP), leading to age-dependent progressive paralysis, neurodegeneration and synaptic damage [56, 65]. Expression of mutant *TARDBP* in zebrafish embryos induced motor neuron degeneration and movement deficits, which were rescued by co-expression of the WT human *TARDBP* gene, suggesting the functional importance of TDP-43 and the potential for pathogenic mutations to cause loss-of-function and toxicity function [66].

#### *Caenorhabditis elegans* and Zebrafish models of C9ORF72 mutation

The ALS/FTD-related gene homolog (*alfa-1*) is a *C. elegans* ortholog of *C9ORF72*. Loss of *alfa-1* leads to motor deficits, motor neuron degeneration, and paralysis in *C. elegans* [67]. In addition, *alfa-1* deletion also leads to dysfunction of lysosomal reorganization and degradation of endocytic elements in nematodes, which disrupts lysosomal homeostasis [68]. Knockdown of a zebrafish ortholog of *C9ORF72* results in axonopathy in MNs, cytoplasmic accumulation of TDP-43, and swimming abnormalities [49]. Recently, a stable *C9ORF72* transgenic zebrafish model was constructed, characterized by accumulation of RNA foci and DPR in muscles and the central nervous system (CNS), increased apoptosis, abnormal motor axons, motor deficits,  muscle atrophy, loss of MNs, cognitive impairment, and reduced survival [69, 70].

Studies of neurodegeneration and toxicity associated with ALS may benefit from the *C. elegans* and Zebrafish models. However, it should be recognized that these are very simple organisms with several limitations, including large anatomical differences from the human brain, lack of obvious tissues and organs, wide use of embryonic stages, and inability to perform more informative behavioral analyses. Simulating some pathological features and phenotypes of human ALS disease is difficult.

### *Drosophila melanogaster*

*Drosophila melanogaster* is an easy handling and cost-effective animal model with short lifespan and complete genome sequenced. They are widely used for studying neurodegenerative diseases including ALS [71, 72]. Most of the mutated genes known to be associated with ALS have been modeled in *Drosophila*, and transgenic flies expressing ALS genes can reliably reproduce some ALS phenotypes such as movement disorders, cellular inclusions, and mitochondrial dysfunction [73].

#### *Drosophila melanogaster* model of SOD1 mutation

Several studies have shown that transgenic flies expressing the human *SOD1* gene carrying point mutations (G85R, A4V, G37R, or G41D) exhibit motor neuron dysfunction, dyskinesia, mitochondrial damage, oxidative stress, and pathological SOD1 aggregation [74–76]. One of the important reasons why *Drosophila melanogaster* is a powerful genetic model is the UAS/Gal4 system, which is widely used to overexpress disease-associated human genes in *Drosophila* [77]. The UAS/Gal4-driven expression of WT or ALS-associated forms of *SOD1* (A4V or G85R) in MNs does not alter the lifespan of flies but results in progressive motor function deterioration [74]. Besides, expression of zinc-deficient human SOD1 in *Drosophila* neurons also produces a locomotor defect linked to mitochondrial dysfunction [76]. Human *SOD1* mutations in a *Drosophila* knock-in model also cause neuronal metamorphosis, muscle contraction and decreased survival rates [78].

#### *Drosophila melanogaster* model of FUS mutation

The only *FUS* ortholog in *Drosophila melanogaster* is *cabeza* [70]. *Drosophila* expressing mutant human FUS or Cabeza have phenotypes such as neurodegeneration, impaired photoreceptors, and neuronal complications [79–82]. It has been reported that these phenotypes can be rescued by introducing a WT human *FUS* transgene in fly mutants of *cabeza* [83]. Investigations in *FUS* transgenic flies have shown that human FUS-induced neurotoxicity can be attenuated by inhibiting nuclear export, confirming that nucleoplasmic transport is involved in the pathogenesis of ALS [84, 85]. Several studies on transgenic *Drosophila* have also confirmed that the FUS-induced neurodegeneration is associated with cellular processes such as transcriptional and translational regulation [86], piRNA biogenesis [87], stress granule assembly [88], and Hippo-signaling pathways [89, 90], and further elucidated the complex pathogenesis of ALS.

#### *Drosophila melanogaster* model of TDP-43 mutation

Several studies have reported that overexpression of the *TBPH* gene (fly ortholog of *TARDBP*) in *Drosophila* and overexpression of mutant or WT human TDP-43 affects motility, axonal transport, pupal eclosion, and lifespan [81, 91–94]. Loss of *TBPH* in *Drosophila* also results in motor impairment and shortened lifespan [95]. Interestingly, several potential therapeutic approaches have been identified using the *Drosophila* TDP-43 transgenic model. Modulations of autophagy [96], mitophagy [97], mitochondrial dynamics [98], glucose and lipid metabolism [99], and stress granule dynamics [100, 101] have been reported as beneficial for fly motor behavior and longevity.

#### *Drosophila melanogaster* model of C9ORF72 mutation

*Drosophila melanogaster* has no *C9ORF72* ortholog, so the consequences of *C9ORF72* deletion in *Drosophila* cannot be determined. A *Drosophila* model of *C9ORF72*-associated ALS has been developed by overexpressing the G4C2 repeat RNA to mimick DPR proteotoxicity, and has revealed some important insights into the pathogenesis of *C9ORF72*-ALS. Ectopic expression of expanded G4C2 or toxic dipeptide repeats in *Drosophila* tissues results in motor deficits, abnormalities of the NMJ, and disorganized microphthalmia [102–105]. Recent studies in several *C9ORF72* transgenic *Drosophila* models have shown that different cellular processes contribute to *C9ORF72*-ALS pathogenesis, such as transcription [104, 106], nucleocytoplasmic transport [107, 108], translation [109], and protein degradation [110].

Despite the many advantages of the *Drosophila* model, the major limitations of this organism are the large anatomical differences from human brains and the impossibility of performing more informative behavioral analyses.

### Rodents (mouse and rat)

Transgenic rodents are the most used animal models and provide important insights into pathogenesis. Rodent models have been widely used in ALS research since the first *SOD1* (A4V and G93A) ALS mouse models were developed in 1994 [15]. Although rats are used much less than mice, they also have physiological characteristics like those in humans and the possibilities for genetic manipulation are historically more recent in rats than in mice. Rodent ALS models with ALS-associated mutations are listed in Table 1.

**Table 1:** Mouse models (*SOD1*, *TARDBP*, *FUS* and *C9ORF72*) used for ALS

Gene type (Refs.)	Promoter	Cortical and hippocampal MNL	Cognitive deficit	Gliosis	Paralysis	Mechanism	Cytoplasmic inclusion
*SOD1*-G93A [111, 112]	Human SOD1	ND	Yes	Yes	Yes	GOF	SOD1, vac
*SOD1*-A4V [113]	Human SOD1	No	ND	No	No	ND	None
*SOD1*-D90A [112]	Human SOD1	No	ND	Yes	Yes	GOF	SOD1, vac
*SOD1*-G85R [114, 115]	Human SOD	ND	ND	Yes	Yes	GOF	SOD1, UBI, LBHI
*SOD1*-G37R [116, 117]	Human SOD1	No	ND	Yes	Yes	GOF	SOD1, vac
hSOD1^WT^ [15]	Human SOD1	ND	ND	Yes	Yes	GOF	SOD1, vac
hSOD1^WT^ [118]	Human SOD1	ND	ND	Yes	No	GOF	SOD1, vac, UBI
*TARDBP*-A315T [119, 120]	BAC	ND	Yes	Yes	No	GOF	TDP43, UBI
*TARDBP*-A315T [119, 120]	mPrp	ND	ND	Yes	Yes	LOF	UBI
*TARDBP*-M337V [121]	mThy1	Yes	ND	Yes	Yes	GOF, LOF	TDP43, UBI, Variable
*TARDBP*-M337V [121, 122]	mPrp	Yes	Yes	ND	No	GOF, LOF	TDP43
*TARDBP*-Q331K [122, 123]	mPrp	Yes	ND	ND	No	GOF, LOF	Vac
*TARDBP*-Q331K [123, 124]	mPrp	Yes	ND	Yes	Yes	GOF	TDP43, p62, vac, UBI
*TARDBP*-G348C [119, 120]	BAC	ND	Yes	Yes	No	GOF	TDP43, UBI
hTDP43 [125, 126]	BAC, mPrp	ND	ND	Yes	Yes	GOF	TDP43, vac, UBI
*FUS*-R521C [127, 128]	Tau, mPrp	ND	ND	Yes	Yes	GOF, LOF	Diffused FUS
*FUS*-R521G [127, 128]	CAG	ND	ND	Yes	Yes	GOF, LOF	None
*FUS*-P525L [129]	Tau	ND	ND	Yes	Yes	GOF	Diffused FUS
hFUS [31, 130]	Tau, MPrp	No	ND	Yes	Yes	GOF	FUS, UBI
FUS^ΔNLS^ [131–133]	HR	Yes(C), ND(H)	ND	ND	No	GOF	FUS
*C9ORF72* [100–1000] *n* [134]	BAC	No	No	No	No	ND	DPR, RNA foci
*C9ORF72* [500] *n* [135]	BAC	No	No	No	No	ND	DPR), RNA foci
*C9ORF72* [64] *n* [136, 137]	AAV-mediated somatogenesis	Yes(C), ND(H)	Yes	Yes	No	GOF	DPR, RNA foci, TDP43
*C9ORF72* [147] *n* [136, 137]	AAV-mediated somatogenesis	Yes(C), ND(H)	Yes	Yes	No	GOF	DPR, RNA foci, TDP43
*C9ORF72* [100] *n* [138]	self-complementary AAV9	Yes	Yes	No	No	GOF	RNA foci, TDP43
*C9ORF72* [450] *n* [135]	BAC	Yes(C), ND(H)	Yes	No	No	GOF	DPR), RNA foci
*C9ORF72* [500] *n* [139]	BAC	Yes	Yes	Yes	Yes	GOF	DPR, RNA foci, TDP43

*n* Number of C9 repeats, *GOF* gain of function, *LOF* loss of function, *UBI* ubiquitin, *vac* vacuolization, *MNL* motor neuron loss, *LBHI* Lewy-body-like hyaline inclusion, *ND* not described, *HR* homologous recombination; *BAC* bacterial artificial, *DPR* dipeptide-repeat proteins, *AAV* Adeno-associated virus

#### Rodent models of SOD1 mutation

Several *SOD1* transgenic rodent models (G93A, D83G, D85G, D86G, D90A, and G37R, among others) have been constructed based on variants found in ALS patients over the past 28 years. Most are established by overexpressing missense, mutated, or truncated human SOD1 [15, 68, 140–142]. Among them, the *SOD1* G93A model is the most widely used. It reproduces some pathological mechanisms in ALS patients such as abundant cytoplasmic inclusions, NMJ injury and extensive inflammation in the spinal cord with reactive gliosis, and exhibits gender difference in disease progression [143, 144]. However, a major drawback of the *SOD1* G93A mouse model is the absence of motor neuron degeneration in the cerebral cortex, which is one of the main hallmarks in human patients [145]. *SOD1* (D83G) transgenic mice show some motor neuron degeneration in the cerebral cortex, but with no paralytic phenotype in the adulthood [146]. Mice homozygous for the *SOD1* D90A mutation accumulate SOD1 aggregates in the ventral horn of the spinal cord and develop a fatal motor neuron disease that progresses slowly, similar to bladder disturbances observed in human ALS patients [147]. Several other models (D85G, D86G, and G37R) all express high levels of SOD1 aggregates, and share common features such as neuroinflammation, glutamate excitotoxicity, mitochondrial alterations, and defective axonal transport in neurons [148]. Most mutations in SOD1 associated with ALS are generally thought to cause ALS through a gain-of-function mechanism. However, the *SOD1*-knockout mice also develop muscle denervation and mitochondrial oxidative stress over time, suggesting that chronic loss of SOD1 activity may contribute to the disease [20, 21]. These SOD1 mouse models have been used for preclinical evaluation of potential treatments for ALS. While some potential treatments have been able to show benefits in the SOD1 mouse models, translation into clinical trials has been poor. For example, minocycline was able to slow disease in *SOD1* (G37R) mice, but it accelerated disease in human clinical trials in a diverse ALS patient group [149].

Rat models of *SOD1* mutation have also been developed, among which the more studied are *SOD1* G93A and H46R models. They show similar pathological features derived from genetic alterations as described in mice, such as upper and lower motor neuron degeneration [150, 151]. Notably, the *SOD1* G93A mutation causes more aggressive disease than the H46R mutation [152]. Recently, in the *SOD1* G93A rat model, Maggot et al. showed that disrupting the blood-spinal cord barrier directly leads to motor neuron degeneration. Intravenous infusion of healthy mesenchymal stem cells into these rats delayed disease progression, preserved barrier function, increased expression of the neurotrophic factor neurturin, and protected motor neurons [153].

#### Rodent models of TDP-43 mutation

Based on the known variants of *TDP-43* in patients with fALS, approximately 20 TDP-43 mouse models have been established [154]. The earliest transgenic TDP-43 mouse models were generated by overexpression of WT or mutant (A315T and M337V) TDP-43 cDNAs under the prion protein gene promoter [120, 155, 156]. Subsequently, researchers generated transgenic mouse models overexpressing exogenous human TDP-43 based on promoters such as Thy1.2 and Camkllα. These transgenic mice display accumulation of pathological aggregates of ubiquitinated proteins in specific neuronal populations, resulting in abnormalities of early neuronal morphology, gliosis, varying degrees of spinal cord pathology, and progressive paralysis and death [124, 157]. Studies have shown that the phenotypic severity correlates with the expression level of mutant TDP-43, and in those animals with high expression, death usually occurs within a week. To understand whether there is a loss-of-function mechanism in TDP-43-related ALS, researchers generated a knockout mouse model. They found that homozygous knockout led to impaired embryogenesis, while heterozygous knockout did not induce symptoms of neuromuscular disease and preserved normal protein expression, suggesting that TDP-43 has an important function during development [28, 157, 158]. Furthermore, in patients with TDP-43 mutation or certain pathological conditions such as FTLD, nuclear TDP-43 redistributes in the cytoplasm and forms cytoplasmic inclusions [25, 159]. While some mouse models can have minimal levels of cytoplasmic TDP-43 [120, 160, 161], most TDP-43 mutant mice show predominantly nuclear localization of TDP-43 and do not reproduce the critical cytoplasmic mislocalization of TDP-43 [162, 163]. These findings raise concerns about the reliability of these mouse models in studies of ALS pathogenesis.

Overexpression of human WT and mutant M337V TDP-43 has been studied in rats, with early phenotypes of immobility, paralysis, and presexual death. In addition, the rats expressing comparable levels of TDP-43 M337V show a more severe phenotype than those expressing WT TDP-43 after 6 months, suggesting that the mutant TDP-43 protein is more toxic than WT TDP-43 [164].

#### Rodent models of FUS mutation

Like TDP-43, the nuclear depletion of FUS proteins and the formation of toxic aggregates in the cytoplasm are important events leading to ALS pathogenesis [165]. Since the discovery of FUS association with ALS [9, 166], several mouse models with *FUS* knockdown or overexpression of WT and mutant *FUS* have been developed (R521C, R521G, P525L, FUS^ΔNLS^, etc.) [129, 131–133]. All these models have varying degrees of phenotypes such as early neuronal loss, motor deficits, and mild behavioral impairments. Two mutants, *FUS* P525L and R521C, have been reported to cause early-onset and late-onset disease in humans, respectively, and induce NMJ deficits and progressive neurodegeneration in mice at 2 and 1 month of age, respectively. Furthermore, the *FUS* P525L mice exhibit more cytoplasmic FUS than *FUS* R521C mice, suggesting that the frequency of FUS accumulation is directly related to the severity of ALS [167]. The *FUS* R521G mice develop hindlimb clenching, NMJ denervation, muscle atrophy, and mild behavioral disturbances before locomotion loss, and eventually die before age of 1 month due to the loss of locomotor function [130, 167]. Partial cytoplasmic mislocalization of FUS in FUS^ΔNLS^ mice is sufficient to cause several behavioral abnormalities, including locomotor hyperactivity and altered social interaction, which precede motor neuron degeneration [131–133].

In the rat *FUS* R512C model, protein aggregation is observed in the brain and the spinal cord, leading to early neuronal loss, gliosis, and motor dysfunction, and eventually typical FTD phenotypic behaviors such as social restriction and hyperactivity [168, 169]. Transgenic rats can also be generated by intravenous administration of adeno-associated virus vector serotype 9 (AAV9) in adult rats. These rats exhibit progressive motor changes and respiratory dysfunction [170].

#### Rodent models of C9ORF72 mutation

In 2013, researchers developed the first mouse model carrying a mutation in the *C9ORF72* gene [171], which exhibits NMJ damage, apoptosis, cognitive impairment, and motor deficits [138, 171]. The researchers then used bacterial artificial chromosomes to generate a cohort of transgenic mice that carry approximately 500 and 1000 repeats of the G4C2 motif and develop extensive RNA foci and DPRs. However, no behavioral changes or neurodegeneration was observed [135, 172–174]. This supports the hypothesis that RNA foci and dipeptides are insufficient to drive degeneration, although this finding may be only specific to these models and not representative of what happens in humans. Furthermore, the *C9ORF72* transgenic model developed by Liu et al. showed phenotypes such as extensive nuclear and cytoplasmic inclusions in neurons, MN loss, muscle denervation, hindlimb paralysis, and decreased survival [139]. However, the degenerative phenotypes of this model were not reproducible when tested in two independent laboratories [175]. Although repeat expansion has been shown to generate toxic products, mRNAs encoding the C9ORF72 protein are also reduced in affected individuals, and the reduced C9ORF72 has been shown to suppress the repeat-mediated increase in autophagy. These results support a disease mechanism in ALS/FTD resulting from reduced C9ORF72, which may lead to autophagy deficits that synergize with the repeat-dependent increase in toxicity [176]. Repeat expansion reduces *C9ORF72* expression and induces neurodegeneration by two mechanisms: accumulation of glutamate receptors leading to excitotoxicity and impaired clearance of neurotoxic DPRs derived from the repeat expansion [177]. Thus, a combined gain-of-function and loss-of-function mechanism leads to neurodegeneration. Currently, conditional knockout mice with neuron-specific deletion of *C9ORF72* have been generated, but they do not exhibit MN degeneration, overt motor deficits, or reduced survival [178].

Knockdown of the *C9ORF72* gene in rats using CRISPR/cas9 technology has been reported without MN loss and motor deficits. Interestingly, when the rats were treated with low doses of  a glutamic acid analog kainic acid, the release of excitatory neurotransmitters was stimulated, leading to susceptibility of motor neurons to excitotoxicity, motor neuron degeneration, and motor deficits [179]. *C9ORF72* knock-in rats were generated by knockin of 80 G4C2 repeats with human flanking fragments within exon 1a and exon 1b of the rat *C9ORF72* locus. These rat models have reduced C9ORF72 protein expression in several CNS regions and show motor deficits due to motor neuron loss at 4 months of age [180].

Although some rodent models can reproduce protein misfolding and aggregation observed in patient brains, most rodent models cannot fully mimic the symptoms and pathologies of many neurodegenerative diseases, including ALS [120, 181–184]. This phenomenon could be due to the genomic, molecular, and anatomic differences between rodents and humans.

### Canine models

Canine degenerative myelopathy (CDM) is an idiopathic pathology that occurs in specific dog breeds and is thought to be a human *SOD1*-related ALS model due to clinical and molecular similarities. More precisely, CDM is characterized by progressive axonal degeneration, muscle atrophy, astrocytosis, peripheral demyelination, and SOD1 inclusions leading to adult-onset neurodegenerative myelopathy and progressive motor dysfunction [185, 186]. CDM shares some molecular and clinical features with upper MN-dominant forms of ALS, such as disease progression and distribution of lesions [187, 188]. Interestingly, to date, only two missense mutations in SOD1 dismutase, T18S and E40K, have been identified as causative for CDM. Unlike ALS, in which *SOD1* pathogenic variants are inherited as dominant, CDM shows recessive inheritance with reduced penetrance [189, 190]. The T18S and E40K mutations do not disrupt the dismutase domain, but both may induce SOD1 aggregation either by reducing the negative charge repulsion or by forming disulfide-linked enzymatically active dimers, thus supporting the gain-of-function hypothesis for SOD1 toxicity [191]. In addition to the loss of MNs, canine models affected by CDM share some other pathological features with *SOD1*-ALS rodent models and patients, such as oligodendrocyte damage leading to demyelination [192], an increase of arginase 1-expressing microglia in the vicinity of motor neurons [193], and upregulation of CB2 receptors in glia cells that serve as a marker of major cellular and biological responses to disease [194].

Considering the clinical and molecular similarities between idiopathic CDM and *SOD1*-ALS, studies of canine idiopathic CDM may help better dissect the pathological mechanisms of ALS. However, it should be considered that dogs with CDM are often euthanized at an early stage of the disease, and therefore the tissues used for investigation can only provide insight into early disease stages.

### Swine models

The swine model has been widely used to study human disease pathology because of its anatomical, physiological, and biochemical similarities with humans, including high similarities in the genome [195] and neuropsychiatric disease characteristics [196]. Currently, several neurodegenerative diseases have been recapitulated in pigs [197–200], including ALS (TDP-43-related and SOD1-related). Chieppa et al. generated the first ALS pig model expressing G93A *hSOD1* by in vitro culture transfection combined with somatic cell nuclear transfer (SCNT) [201]. The transgenic SOD1^G93A^ pigs exhibit motor neuron degeneration, hindlimb motor deficits, expression of mutated *SOD1* copies, gliosis, and protein aggregation in an age-dependent manner [202, 203]. During early disease stages, the mutant SOD1 does not form cytoplasmic inclusions but instead shows nuclear accumulation and ubiquitinated nuclear aggregates in the SOD1^G93A^ pigs, as in the brains of some ALS patients [202]. At approximately 27 months of age, the transgenic SOD1^G93A^ pigs undergo a prolonged symptomatic phase characterized by increased amounts of total TDP-43 in peripheral blood mononuclear cells. Severe skeletal muscle pathology, including inflammation and necrosis, is observed in the late stages of the disease [203]. Subsequently, Wang et al. also used SCNT to generate the first M337V TDP-43 transgenic pig model exhibiting severe phenotypes and early death. TDP-43 aggregates were detected in the cytoplasm of the spinal cord and brain neurons in the M337V TDP-43 transgenic pigs. The M337V TDP-43 protein alters neuronal RNA splicing by interacting with NeuN-associated protein-associated RNA splicing factors, as reported in ALS patients [204]. Convincingly, with gene silencing approaches such as viral-mediated delivery of shRNAs, large animal models such as pigs could be widely used in drug development and drug safety studies [205, 206].

Although the pig model can recapitulate some pathological characteristics of neurodegenerative diseases, there are still some limitations, such as high cost, long growth cycle, and lack of systematic behavioral and cognitive testing systems.

### Non-human primate models

The brains of non-human primates are evolutionarily closest to the human brain with many structural, cognitive, and functional features. The brains of non-human primates have a complex network of brain connections involving the neocortex and prefrontal cortex, thus enabling the development of higher brain functions such as thinking, learning, decision-making, and judgment [207, 208]. At the molecular level, the brains of non-human primates share more similar gene expression patterns with human brains than mouse brains do [209]. These features, along with neuroanatomical and genetic similarities, make non-human primates highly desirable models for neurodegenerative diseases, including ALS. In 2012, Uchida et al. created TDP-43-overexpressing cynomolgus monkeys by injecting adeno-associated virus (AAV)-based human WT TDP-43 coding sequences into the C5-C6 spinal segment of cynomolgus monkeys [210]. After 2–3 weeks, the monkeys exhibited progressive motor weakness and muscle atrophy with fasciculations in the muscles of the distal hand on the injection side; complete paralysis of the ipsilateral hand was observed 2–5 weeks after onset. At the same time, symptoms such as muscle atrophy and weakness also appeared in the contralateral hand. At the cellular level, diffuse mislocalization of TDP-43 in the cytoplasm was evident in α-MNs, but accumulation was infrequent, suggesting that this model does recapitulate some of the clinical features of ALS patients as well as pathological features in the spinal cord. In subsequent years, Borel et al. obtained marmoset and macaque *SOD1*-ALS models by intrathecal delivery of AAV encoding an artificial SOD1-specific microRNA and determined reduced SOD1 levels in motoneurons and spinal cord slices [211, 212]. Using the same technique, stereotaxic injection of *FUS*-targeting shRNA in *Callithrix jacchus* was used to generate a *FUS*-ALS marmoset model [213]. It is undeniable that gene silencing methods using viral delivery can play an important role in drug development and drug safety research. However, these monkeys were only manipulated with gene silencing to simulate the loss of *SOD1* and *FUS* in the neurons of ALS patients and were not studied for neuropathological characteristics and behavioral phenotypes. Recently, to investigate the subcellular distribution of mutant TDP-43 in the monkey brain, Yin et al. injected a viral vector expressing mutant TDP-43 (M337V) directly into the substantia nigra of rhesus monkeys. Three months after injection, all the monkeys with TDP-43 injection developed significant left upper-extremity weakness at 2–4 weeks post-injection, and the severity increased and stabilized 3–4 months later [214]. Most of the mutant TDP-43 was distributed in the cytoplasm of the monkey brain, which is consistent with the cytoplasmic distribution of TDP-43 in the brains of ALS patients and the spinal cords of monkeys overexpressing WT TDP-43. Notably, non-human primate-specific caspase-4, but not the mouse homolog caspase-11, removes the nuclear localization signal-containing N-terminal domain, leading to accumulation of fragmented TDP-43 in the cytoplasm [210, 214]. The cleavage of TDP-43 mediated by the primate-specific caspase-4 provides additional clues to the cytoplasmic TDP-43-mediated gain-of-toxicity and points to potential therapeutic strategies to prevent or reduce TDP-43-associated neuropathology.

Non-human primate models have anatomical, physiological, and biochemical characteristics relevant to humans, including high genomic similarity. However, similar as pig models, the non-human primate models of neurodegenerative diseases have limitations of high cost and ethical issues. Table 2 lists large animal models of ALS with ALS-associated mutations.

**Table 2:** Large animal models (*SOD1*, TDP-43, *FUS* and *C9ORF72*) used for ALS research

Species [Refs.]	Genetic anomaly	Modification approach	Pathology and phenotypes
Monkey [214, 215]	TDP-43 (M337V)	Brain regional expression of transgenic TDP-43	Cytoplasmic accumulation of mutant TDP-43, motor function deficits
Monkey [210]	TDP-43 (WT)	Spinal cord expression of human WT TDP-43	Progressive motor weakness and muscle atrophy. Cytoplasmic mislocalization of TDP-43
Monkey [211, 212]	*SOD1*	Intrathecal delivery of AAV encoding *SOD1*-specific microRNA	Not described
Monkey [213]	*FUS*	Injection of *FUS*-targeted shRNA	Not described
Pig [204]	TDP-43 (M337V)	Embryonic expression of transgenic TDP-43	Severe phenotypes and early death
Pig [202]	*hSOD1* (G93A)	Embryonic expression of transgenic *SOD1*	Hind limb movement deficits, loss of motor neurons, formation of neuronal intranuclear inclusions in early disease stage
Pig [201]	*hSOD1* (G93A)	Embryonic expression of transgenic *SOD1*	No ALS-like phenotypes
Canine [185, 186]	*SOD1* (T18S, E40K)	Spontaneous	Progressive axonal degeneration, muscle atrophy, and SOD1 inclusions

## Challenges and future directions

Effective therapeutics for ALS are urgently needed, but for drug applications, translating results from small animal models to human clinical trials remains limited and challenging. This may be due to the genomic, molecular, and anatomical differences between small animals and humans. For instance, in rodents, some genetic mutation models show a milder ALS phenotype than humans, while some models show no signs of neurodegeneration. Rodents typically live for less than 3 years, a short lifespan that may not be long enough for the occurrence of neurodegeneration that typically takes decades to occur in humans. Furthermore, only about a quarter of alternatively spliced exons for a given transcript is conserved between humans and rodents [216]. The importance of this difference in the pathogenesis of ALS remains unexplored. These factors may lead to substantial failures in the translation from preclinical animal studies to effective clinical treatments for ALS.

**Fig. 2:**
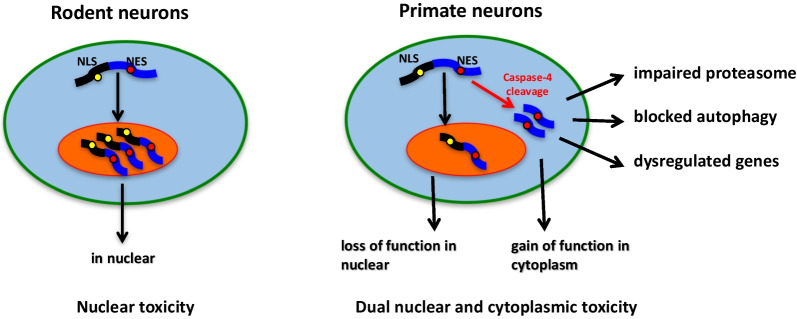
New pathogenic insights from monkey models expressing mutant TDP-43. TDP-43 remains in the nucleus of rodent neurons to elicit nuclear toxicity. In the primate neurons, however, the primate-specific caspase-4 cleaves TDP-43 to produce truncated TDP-43, which redistributes in the cytoplasm, resulting in cytoplasmic toxicity

The current large animal models have demonstrated important species-dependent differences in neuropathology. They serve as important tools to study pathogenesis and pathological events that may uniquely occur in humans and thus could serve as new therapeutic targets. For example, the cytoplasmic distribution of mutant TDP-43 (M337V) in rhesus monkey and pig brains highlights the value of large animal models for studying the cytoplasmic toxicity of TDP-43 [204, 210, 214]. Thus, the selective expression of modifiers or targets of disease proteins in large animal models contributes to specific neuropathological events, which may not occur in small animal models. Given these factors, it is expected that more and more large animals will be accepted as models of choice for research on human diseases and translational medicine. In addition, the important information gained from large animal models is invaluable for generating more humanized mouse models. However, there are barriers to generating large animal models, such as the high cost of animals, the current inefficiency of gene targeting in large animals, and the large amount of time required, which have hindered the widespread use of such animal models for research (Fig. 2).

## Conclusions

In this review, we describe several commonly used model organisms (worms, flies, zebrafish, rodents, dogs, pigs, and non-human primates) as ALS models. Undoubtedly, the models summarized here play a pivotal role in uncovering the myriad cellular and molecular determinants involved in ALS and its progression, and in showing the multifactorial and non-cell-autonomous nature of the disease. To understand the causes and mechanisms of ALS, simultaneous and comparative analyses of these animal models are required while keeping in mind the limitations of these models. Overall, this review describes the pros and cons of various ALS models as well as common features and differences, highlights insights unique to large animal models of ALS, and discusses the use of important pathogenic insights from large animal models to advance the use of rodent models to simulate important pathological features of ALS.

## Data Availability

Not applicable.

## Abbreviations

ALS: Amyotrophic lateral sclerosis
SOD1: Superoxide dismutase-1
C9ORF72: Chromosome 9 open reading frame 72
FUS: Fused in sarcoma
TARDBP: TAR DNA-binding protein
FTLD: Frontotemporal lobar degeneration
DPR: Dipeptide repeat protein
NMJ: Neuromuscular junction
CNS: Central nervous system
AAV: Adeno-associated virus
CDM: Canine degenerative myelopathy
SCNT: Somatic cell nuclear transfer
MN: Motor neuron
